# YLT-11, a novel PLK4 inhibitor, inhibits human breast cancer growth via inducing maladjusted centriole duplication and mitotic defect

**DOI:** 10.1038/s41419-018-1071-2

**Published:** 2018-10-18

**Authors:** Qian Lei, Lu Xiong, Yong Xia, Zhanzhan Feng, Tiantao Gao, Wei Wei, Xuejiao Song, Tinghong Ye, Ningyu Wang, Cuiting Peng, Zhongping Li, Zhihao Liu, Luoting Yu

**Affiliations:** Lab of Medicinal Chemistry, State Key Laboratory of Biotherapy and Cancer Center, West China Hospital, Sichuan University and Collaborative Innovation Center for Biotherapy, 610041 Chengdu, China

## Abstract

Polo-like kinase 4 (PLK4) is indispensable for precise control of centriole duplication. Abnormal expression of PLK4 has been reported in many human cancers, and inhibition of PLK4 activity results in their mitotic arrest and apoptosis. Therefore, PLK4 may be a valid therapeutic target for antitumor therapy. However, clinically available small-molecule inhibitors targeting PLK4 are deficient and their underlying mechanisms still remain not fully clear. Herein, the effects of YLT-11 on breast cancer cells and the associated mechanism were investigated. In vitro, YLT-11 exhibited significant antiproliferation activities against breast cancer cells. Meanwhile, cells treated with YLT-11 exhibited effects consistent with PLK4 kinase inhibition, including dysregulated centriole duplication and mitotic defects, sequentially making tumor cells more vulnerable to chemotherapy. Furthermore, YLT-11 could strongly regulate downstream factors of PLK4, which was involved in cell cycle regulation, ultimately inducing apoptosis of breast cancer cell. In vivo, oral administration of YLT-11 significantly suppressed the tumor growth in human breast cancer xenograft models at doses that are well tolerated. In summary, the preclinical data show that YLT-11 could be a promising candidate drug for breast tumor therapy.

## Introduction

Breast cancer is the second most common cancer among women worldwide; it is the fifth most common cause of death from cancer in women. The incidence of this disease in China is also growing rapidly and is estimated to reach 2.5 million cases by the end of year 2021^1–3^. Despite intensive efforts have been made, there is still no satisfied target drug to relieve the tumor burden and prognosis^4,5^.

Many antitumor agents dampen malignant growth by disturbing the mitotic progression^6^. The polo-like kinases (PLKs) are identified as a family with essential roles in mitosis, including mitotic entry, spindle formation, centrosome duplication, and cytokinesis^7–10^. Among this family, PLK4 (also called Sak) is the most structurally divergent polo family member, which only contains one polo-box domain in the C-terminal noncatalytic region^11,12^. PLK4 is localized to centrosome throughout the cell cycle and tightly controls the centrioles duplication so that mitosis can proceed correctly^13,14^. Overexpression of PLK4 is frequently detected in many metastatic human cancers and connected with cancer progression or poor prognosis^15–20^. Besides, in comparison to normal mice, the PLK4 haploinsufficent mice truly appear to be a higher possibility in tumorigenesis^15,21^. Suppressing PLK4 activity leads to loss of centrosome numeral integrity and spindle malformation or disorientation. These results could accelerate the formation of aneuploidy/polyploidy and chromosomal instability, which makes tumor cells more prone to disorder during the late mitotic progression, ultimately causing mitotic catastrophe and cell death^22–25^. Extensive studies in the past decade have demonstrated that PLK4 is dysregulated in human breast cancer as well as other cancers^18^. Furthermore, combining RNA interference screening with gene expression analysis in human breast cancer cell lines identifies that the activity of PLK4 is crucial for human breast cancer proliferation^18,26,27^. Therefore, PLK4 may be a promising therapeutic target for the human breast cancer therapeutics. However, to date, studies about PLK4 inhibitors are limited^28–31^, and there is only one small-molecule PLK4 inhibitor under clinical trial.

In this work, we described a novel small-molecule PLK4 inhibitor identified from our compound libraries, YLT-11, of which the antineoplastic activity was evaluated both in vitro and in vivo. In vitro, YLT-11 inhibited the proliferation of breast cancer cell lines, especially  for triple-negative breast cancer (TNBC) cells in a concentration-dependent and time-dependent manner. Moreover, YLT-11 interfered with centriole duplication by targeting PLK4 kinase activity, further resulting in the defect of mitotic checkpoint function, abortive mitosis, endoreduplication, and aneuploidy, which finally induced cell death. In vivo, YLT-11 exerted satisfactorily antineoplastic activity in three breast tumor models. Besides, YLT-11 also showed a good safety profile in the sub-acute toxicity test. Taken together, our results indicate that YLT-11 could be a new potent candidate for treatment of breast cancer that is considered worthy of further evaluation.

## Results

### Knocking down PLK4 expression inhibits cancer cell proliferation

**Fig. 1:**
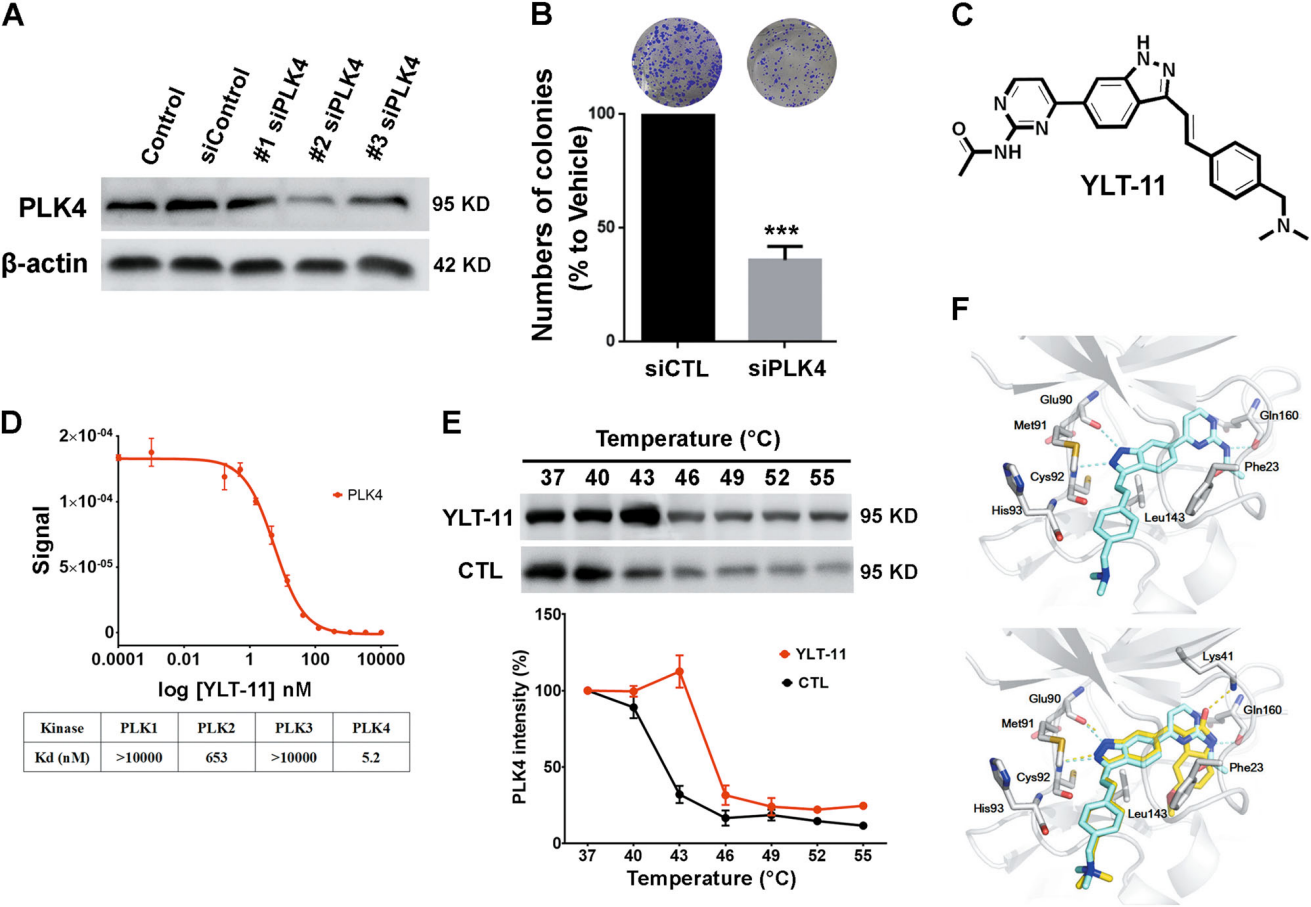
The activity of PLK4 could be potently inhibited by YLT-11, a novel and specific PLK4 inhibitor. **a** MDA-MB-231 transfected with PLK4 siRNA. The expression of PLK4 was determined by Western blotting. **b** The number of colonies in MDA-MB-231 transfected with PLK4 siRNA. Quantification was shown in the lower panel. Data are expressed as mean ± SD for three independent experiments. Columns, mean, bars, SD (***p < 0.001 vehicle control). **c** Chemical structure of YLT-11. **d** Binding assays for YLT-11–PLK4 interaction. Upper panel: Determination of quantitative binding constants of PLK4; lower panel: binding constants of PLK family. **e** Cellular thermal shift assay from 40 to 55 ℃ of MDA-MB-231 lysates with or without YLT-11 incubation. The image (upper panel) and quantification of the band intensities (lower panel) in immunoblotting. Graphic data were run in triplicate and shown as the mean ± SD. **f** YLT-11 is docked into the active site of PLK4, showing interactions between YLT-11 and PLK4 in the three-dimentional structure, and YLT-11 binds to PLK4 via a mode highly similar to that of the inhibitor 400631

To study the effects of PLK4 on breast cancer cells proliferation, three independent small interfering RNAs (siRNAs) specific to PLK4 were designed and transfected into MDA-MB-231 cells. The efficiency of siRNA in silencing PLK4 expression was determined by Western blot (Fig. 1a and Supplementary Fig 1A). The effects of knocking down PLK4 on cell proliferation were then conformed by colony formation assay and MTS assay. These results indicated that the proliferation of cancer cells conspicuously decreased in a manner dependent on the expression of PLK4 (Fig. 1b and Supplementary Fig 1B), suggesting that PLK4 played an important role in the growth of breast cancer cells. Furthermore, quantitative reverse transcription polymerase chain reaction was used to compare PLK4 messenger RNA levels in breast cancer cell lines to the normal mammary cell, and the results demonstrated that PLK4 levels are significantly higher in breast cancer cells (Supplementary Fig 1C).

### Kinase inhibition profile and molecular models of YLT-11

Our group dedicated their efforts to designing and synthesizing numerous PLK4 inhibitors, which has been reported before^32^. Among the candidates, the small-molecule YLT-11, which was characterized by a (*E*)-4-(3-arylvinyl-1*H*-indazol-6-yl)pyrimidin-2-amine skeleton (Fig. 1c), was chosen as the lead compound for its preferable activity on PLK4. As is shown in Supplemental Table 1, YLT-11 exhibited inhibitory activity on PLK4 with a half maximal inhibitory concentration (IC_50_) value of 22 nM. Meanwhile, good selectivity of YLT-11 for PLK4 was also evident, compared with other kinases in this family or a panel of mitotic kinases, including JNK, TOPK, ERK, and so on. In competition binding assay, a strong binding affinity between PLK4 and YLT-11 was identified, with a binding constant (*K*_d_) of 5.2 nM (Fig. 1d). In addition, the thermal shift assay was also used to verify YLT-11 and PLK4 interaction, and the results indicated that YLT-11 increased the thermal stability of PLK4 (Fig. 1e). Overall, these data indicated that YLT-11 was most the active against PLK4, which is likely to be its primary target.

We further analyzed the interaction between YLT-11 and PLK4 kinase domain. Molecular docking showed that YLT-11 was likely to bind in the ATP-binding pocket of PLK4, and hydrogen-bonding interactions of YLT-11 with Lys41, Thr159, and the catalytically important residues Cys92 and Glu90 on the linkage region were observed, while hydrophobic interactions with Val26, Leu143, and Phe23 residues and π–π stacking interactions with Phe23 were also presented (Fig. 1f). The results suggested that YLT-11 would be an ATP-competitive PLK4 inhibitor.

### Antiproliferation activities of YLT-11 against human breast cancer cell lines

**Fig. 2:**
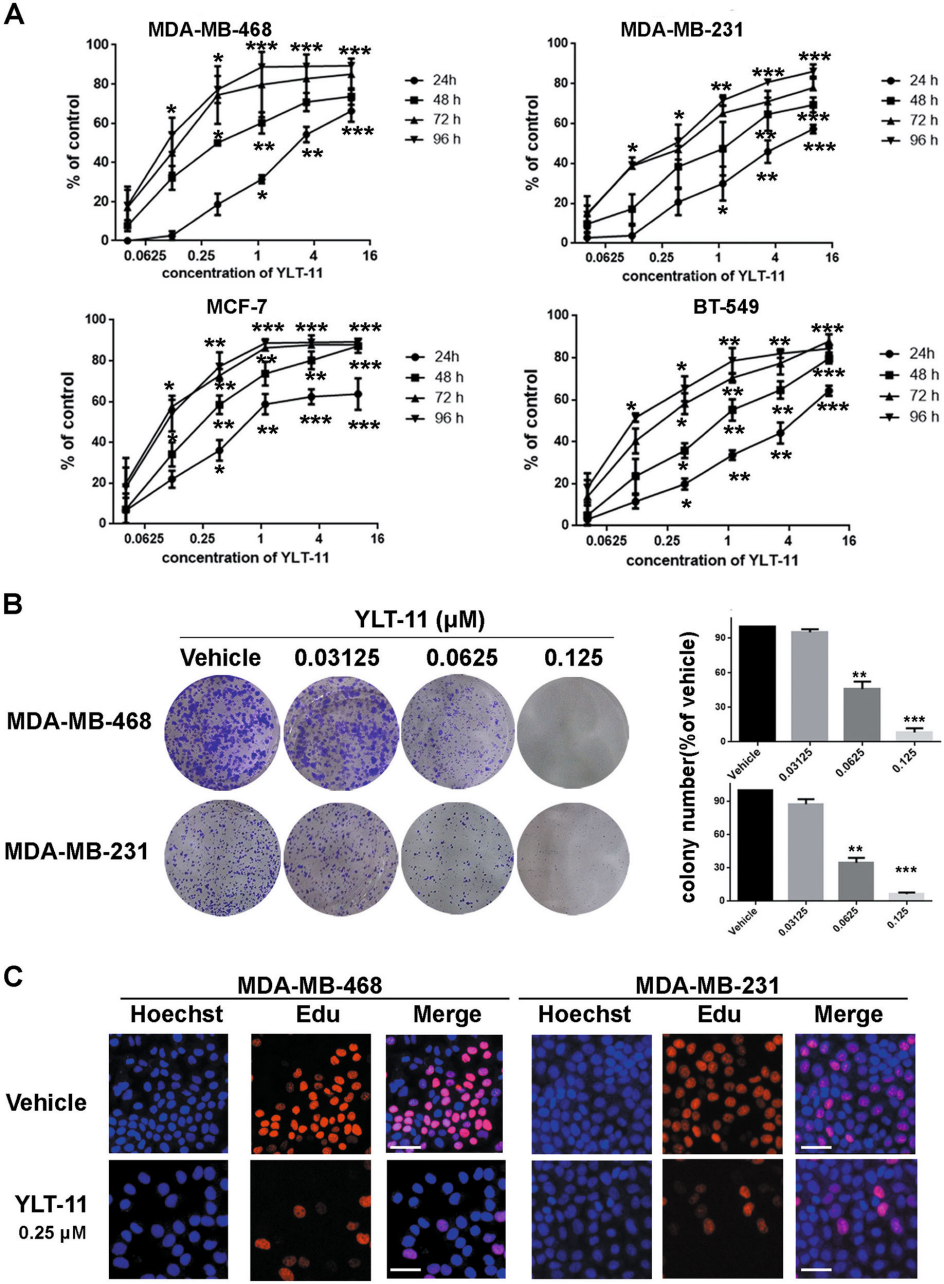
Antiproliferative activities of YLT-11 against human breast cancer cell lines. **a** Four different breast cancer cells were treated with increasing doses of YLT-11 for 24, 48, 72, and 96 h, respectively. Each point represents the mean ± SD for three independent experiments (*p < 0.05, **p < 0.01, ***p < 0.001 vs. vehicle control). ** b** Effects of YLT-11 on cell colony formation after treatement for 2 weeks. Quantification is shown in the right panel. Columns, means (*n* = 3); bars, standard deviation (*p < 0.05, **p < 0.01, ***p < 0.001). **c** YLT-11 inhibited MDA-MB-468 and MDA-MB-231 cell proliferation. The EdU incorporation assay was examined on cells after exposure to YLT-11 for 24 h. EdU-positive (marked by red fluorescent staining) and Hoechst 33342 staining (marked by blue fluorescent staining) cells represented the proliferating and total cells, respectively. Images shown are representatives of three independent experiments. Scale bars, ×20 for micrograph

A panel of 12 human breast cancer cells were screened and results showed that YLT-11 significantly decreased the viability of different subtypes of breast cancer cells (Supplemental Table 2), especially  for the TNBC cell lines in which IC_50_ values ranged from 68 to 120 nM. Then, we chose four cancer cells (MDA-MB-231, MDA-MB-468, BT549, and MCF-7) that were sensitive to YLT-11 for further experiment. As shown in Fig. 2a, after treatment with YLT-11 for indicated time, proliferation of these cancer cells were substantially inhibited by the escalating doses of YLT-11. However, YLT-11 only exhibited negligible inhibitory activity to the normal mammary cells MCF-10A, with an IC_50_ value over 10 μM (Supplemental Table 2). These results indicated that YLT-11 could inhibit the proliferation of breast cancer cells in a time-dependent and concentration-dependent manner. Moreover, the colony formation assay showed that YLT-11 markedly inhibited the colony numbers (Fig. 2b), and the Edu incorporation assays demonstrated that YLT-11 potently suppressed the DNA replication of cancer cells (Fig. 2c and Supplementary Fig 2). Collectively, these results demonstrated that YLT-11 could substantially suppress the breast cancer cell proliferation in vitro.

### YLT-11 induced aberrant centriole duplication in breast cancer cells

**Fig. 3:**
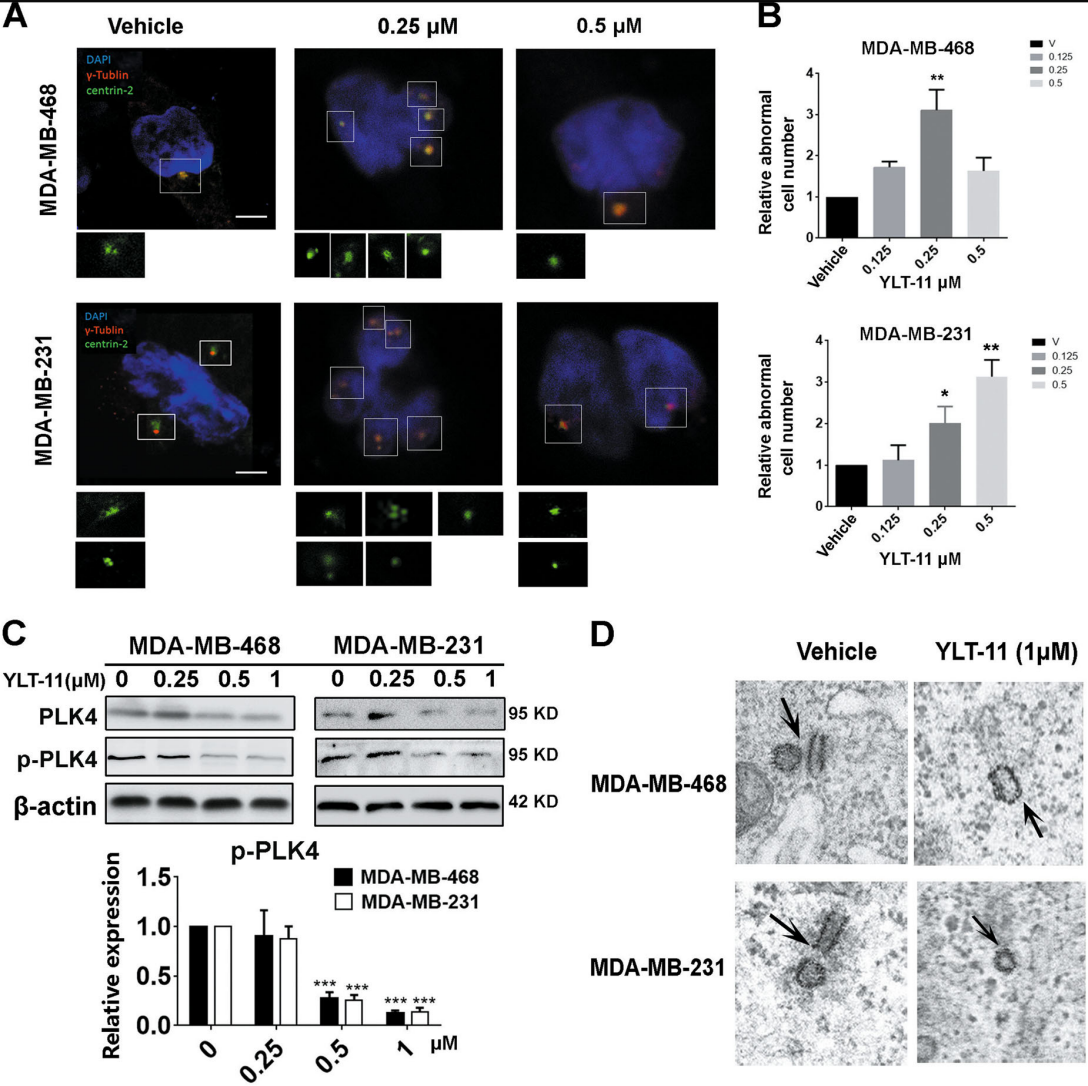
YLT-11 induced aberrant centriole duplication in breast cancer cells. **a** MDA-MB-468 and MDA-MB-231 were treated with YLT-11 with an increasing concentration for 24 h, and the centrioles and centrosomes were determined by immunostaining for centrin 2 (green fluorescent staining) and γ-tublin (red fluorescent staining), respectively. DNA was marked by DAPI. Insets show higher magnification views of the centrioles at the spindle pole (boxed regions). Scale bars, 5 μm; 1 μm for insets. **b** The relative number of cancer cells with abnormal centrioles after treatement with YLT-11 for 24 h, compared with the vehicle group, were counted. Columns, means (*n* = 3); bars, standard deviation (*p < 0.05, **p < 0.01). **c** Expression of p-PLK4 was determined by Western blotting. MDA-MB-468 and MDA-MB-231 were treated with DMSO or the indicated concentrations of YLT-11 (0.25, 0.5, and 1 μM) for 48 h, and the expressions of p-PLK4 and PLK4 were detected with a specific antibody. Each has the expression of β-actin as the internal control. Protein expression was quantified by densitometry analysis using ImageJ and normalized against β-actin expression. Columns, mean; bars, SD, *p < 0.05. **d** Electron micrographs showed the number of centrioles in cells treated with 1 μM YLT-11 for 48 h (right panels), whereas the DMSO group showed only two centrioles (right panel). Scale bar, 500 nm

In eukaryotic cells, the centrosome is duplicated in a semiconservative manner to insure the fidelity of the cell division process^33^. Previous studies have proved that centriole duplication is regulated by PLK4, thus we further investigated the effect of YLT-11 on centriole numbers using dual indirect immunofluorescence labeling. Cancer cells were treated with YLT-11,  then centrioles and centrosomes were determined by immunostaining for centrin 2 and γ-tublin, respectively. As shown in Fig. 3a, b, after exposure to YLT-11 at various concentrations for 24 h, the amount of cells with abnormal centriole numbers were increased with the rising concentration of YLT-11. At low concentrations (≤0.25 μM), YLT-11 increased the centriole numbers, whereas the centriole duplications were conspicuously suppressed when the concentration reached 0.5 μM, which resulted in each spindle pole in cells only containing one single centriole. According to previous research, this bimodal effect on centriole duplication might be due to the different degrees of activity inhibition of PLK4 by YLT-11.

Centriole duplication is under the control of PLK4 and its stability is directly linked to the activity of the enzyme, with active PLK4 phosphorylating itself to promote its own destruction^34^. Previous reports have identified that partial inhibition of PLK4 activity could elevate the levels of PLK4, subsequently leading to overduplication of centrioles. Thus, we examined the effect of YLT-11 on PLK4 activity. As shown in Fig. 3c, YLT-11 effectively inhibited PLK4 phosphorylation with the increase in concentration, which was consistent with the effect on centriole duplication (Fig. 3a, b), indicating that the overduplication of centrioles observed at lower concentrations of YLT-11 might result from an increase in protein levels of partially active PLK4. Additionally, the pictures captured by electron microscopy, which was independent of antibody localization, revealed that centriole duplications were inhibited apparently after treatment with 1 μM YLT-11 (Fig. 3d). Taken together, these data indicated that the activity of PLK4 was inhibited by YLT-11, which resulted in the aberration of centriole duplication.

### YLT-11 induced mitotic defects and disturbed mitotic checkpoint function

**Fig. 4:**
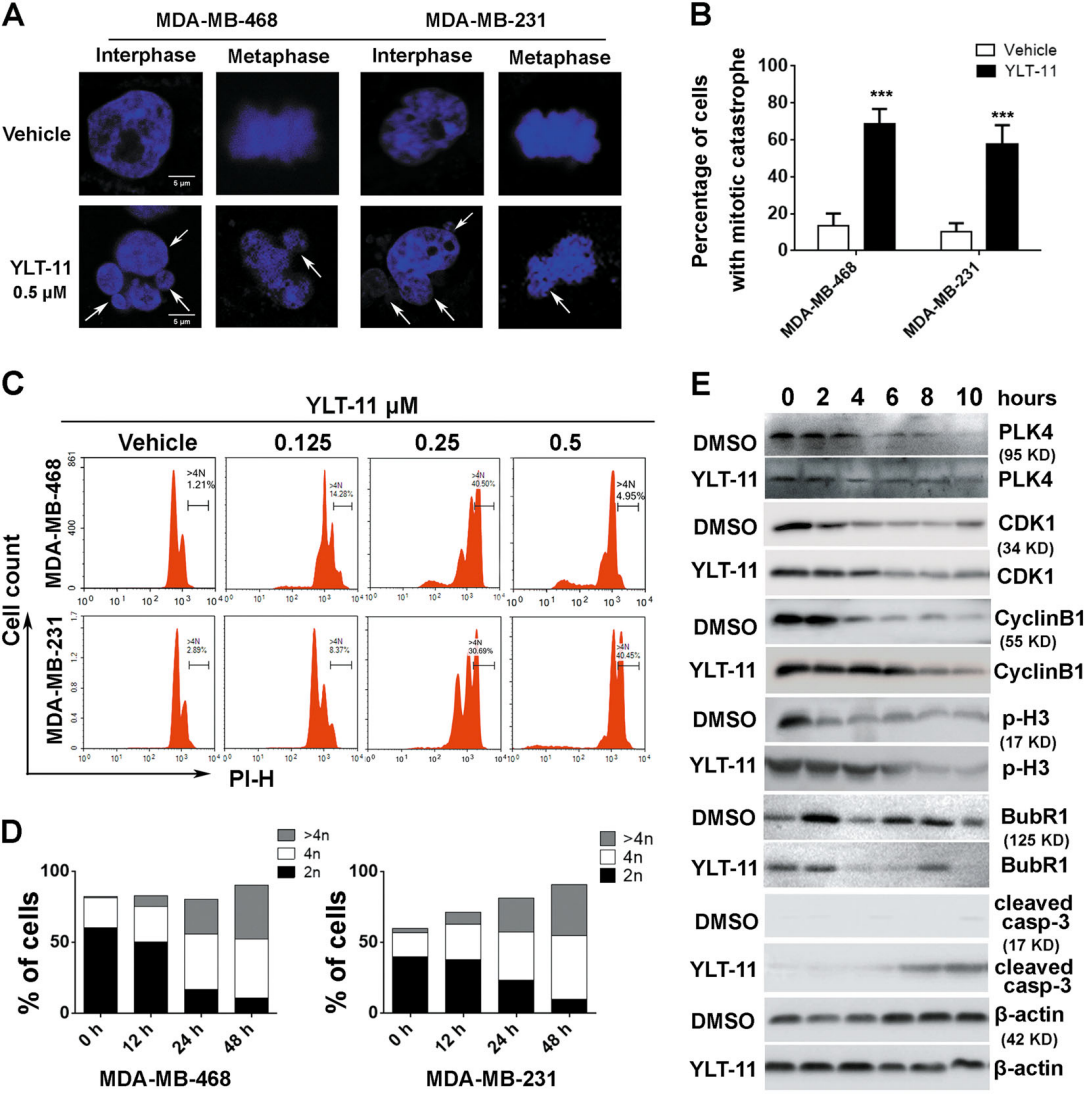
YLT-11 induced mitotic defects and disturbed mitotic checkpoint function. **a** MDA-MB-468 and MDA-MB-231 cells were treated with DMSO or 0.5 μM YLT-11 for 30 h and then stained with DAPI to detect the karyomorphism in different stages. Multinucleation, micronuclei, and chromosome mal-disjunction were marked by arrowheads. **b** Percentage of cells with mitotic catastrophe is quantified. Columns, means (*n* = 3); bars, standard deviation, (***p < 0.001). **c** Breast cancer cell lines were treated with an increasing concentration of YLT-11 for 30 h and stained with propidium iodide prior to analysis of DNA content by flow cytometry. Gating (>4*N*) indicated the percentage of aneuploid/polyploid cells. **d** Breast cancer cell lines were treated with YLT-11 (0.25 μM) for indicated times. Percentage of cancer cells in different stages. 2*N*, 4*N*, and >4*N* presented G1, G2/M, and aneuploid/polyploid cells, respectively. **e** Western analyses of key mitotic checkpoint protein. MDA-MB-468 cells were synchronized by nocodazole for 16 h and allowed to proceed in the presence or absence of 0.5 μM YLT-11. β-actin served as a loading control

As mentioned before, abnormal centriole duplication caused aberrant mitoses, eventually leading to cell arrest and cell death. Therefore, we continued to investigate the possible effect of YLT-11 on mitosis. As shown in Fig. 4a, cancer cells treated with YLT-11 displayed a series of characteristics about mitotic catastrophe, including multinucleation, micronuclei, and chromosome mal-disjunction. Then, the cells were quantified, and approximately 67% of treated cells demonstrated the sign of mitotic catastrophe in MDA-MB-468, and in MDA-MB-231, the value is about 58% (Fig. 4b).

We further detected the impact of YLT-11 on DNA content of individual cancer cell. As shown in Fig. 4c, d, YLT-11 evoked the accumulation of tumor cells with ≥4*N* DNA content in a concentration-dependent and time-dependent manner. The cell percentages in G_2_/M (4*N*) phase increased, whereas the cell percentages in G_1_ (2*N*) phase decreased. Meanwhile, the polyploidy/aneuploidy (>4*N*) were also increased, suggesting that DNA replication continued to occur in the absence of cytokinesis. And more remarkable, at high concentration, the accumulated aneuploid cells collapsed finally with significant cell death in MDA-MB-468, indicating that cancer cells became polyploid or aneuploid as the mitotic catastrophe that occurred would eventually trigger mitotic death.

Mitotic defect was induced in several different ways, including impeding a complex correction between cell cycle kinase (CDK1 and PLK family) and core mitotic checkpoint component^35^. To check the possible molecular mechanism of YLT-11, we next examined the effects of YLT-11 on mitotic proteins. MDA-MB-468 cells were synchronized at the G2/M border with the help of nocodazole and allowed to traverse mitosis in the presence or absence of YLT-11 (Fig. 4e). Analysis of the data revealed that treatment of cells with YLT-11 resulted in a drastic reduction of PLK4 at all stages of cell cycle, demonstrating that YLT-11 acts as a potent inhibitor of PLK4. Moreover, compared with the control group, the cells in the treated group delayed their mitotic phase through extending the accumulation time of cyclinB1, CDK1, and p-H3. Whereas the mitotic checkpoint protein BubR1, which was a substrate of PLK4, was downregulated significantly at the 2 h and later time points, indicating that the aberrant mitotic cells had slipped out of mitosis without cytokinesis and formed multinucleated, pseudo G1-like aneuploid cells (Fig. 4c, d). In addition, apoptosis induction was confirmed by detection of cleaved caspase-3 at 8 h post release. These observations indicated that YLT-11 disturbed the mitotic checkpoint function and resulted in endoreplication, followed by massive cell death.

### YLT-11 induced apoptosis of breast tumor cells in vitro

**Fig. 5:**
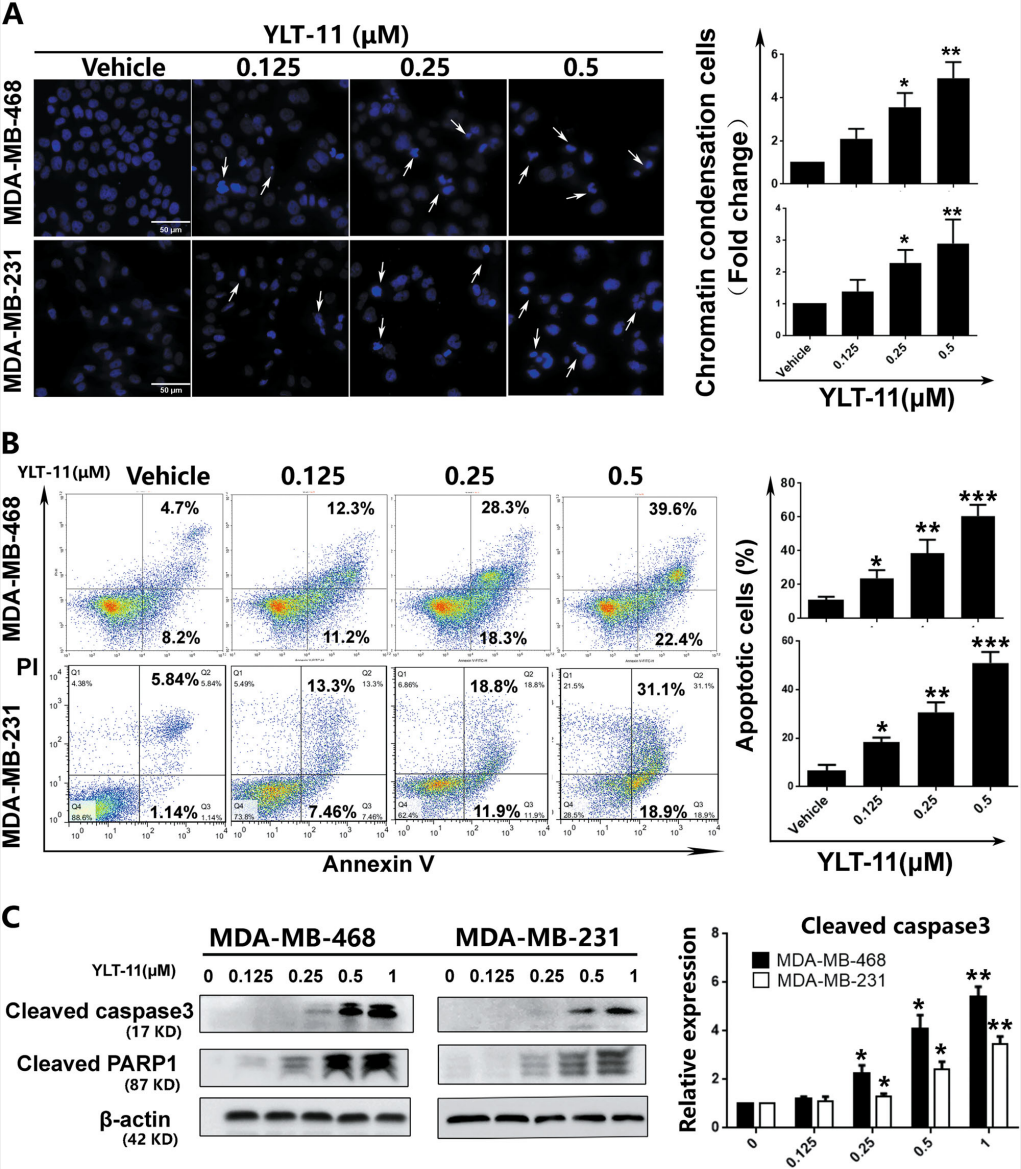
YLT-11 induced apoptosis of breast cancer cell lines. **a** Cell morphological alterations and nuclear changes of MDA-MB-468 and MDA-MB-231 cells were analyzed by staining with Hoechst 33342 (10 mg/mL) and visualized using a microscope after treatment with increasing doses of YLT-11 for 24 h. **b** MDA-MB-468 and MDA-MB-231 cells were treated with YLT-11 for 48 h and were detected by flow cytometry after Annexin V/PI staining. **c** The expressions of cleaved caspase-3 and cleaved PARP1 were determined via western blotting. Protein expressions were quantized by densitometry analysis using ImageJ (shown in the right panel). Data are expressed as mean ± SD for three independent experiments. Columns, mean; bars, SD (*p < 0.05, **p < 0.01, ***p < 0.001)

The cell cycle and Western blot analysis showed that the decrease of aneuploidy was accompanied by massive cell death (Fig. 4c, e). We thus further substantiated the fate of aneuploid cancer cells upon continuous exposure to YLT-11. Morphological changes belonging to apoptotic cells containing cell shrinkage and chromatin condensation (brighter-blue fluorescent pointed by arrowheads) were observed in tumor cells treated with YLT-11 (Fig. 5a). Meanwhile, the Annexin V/propidium iodide (PI) staining was also confirmed by the pro-apoptotic effect of YLT-11 on breast tumor cells, as shown in Fig. 5b and Supplementary Fig 3; YLT-11 induced apoptosis of cancer cells in a time-dependent and concentration-dependent manner. Furthermore, the expression of cleaved caspase-3 and cleaved PARP1 increased in these cancer cells after exposure to YLT-11 (Fig. 5C). Together, these data substantiated that YLT-11-induced cell death is driven, at least in part, by apoptosis.

### Antitumor efficacy and mechanisms of action of YLT-11 in breast tumor xenograft models in vivo

**Fig. 6:**
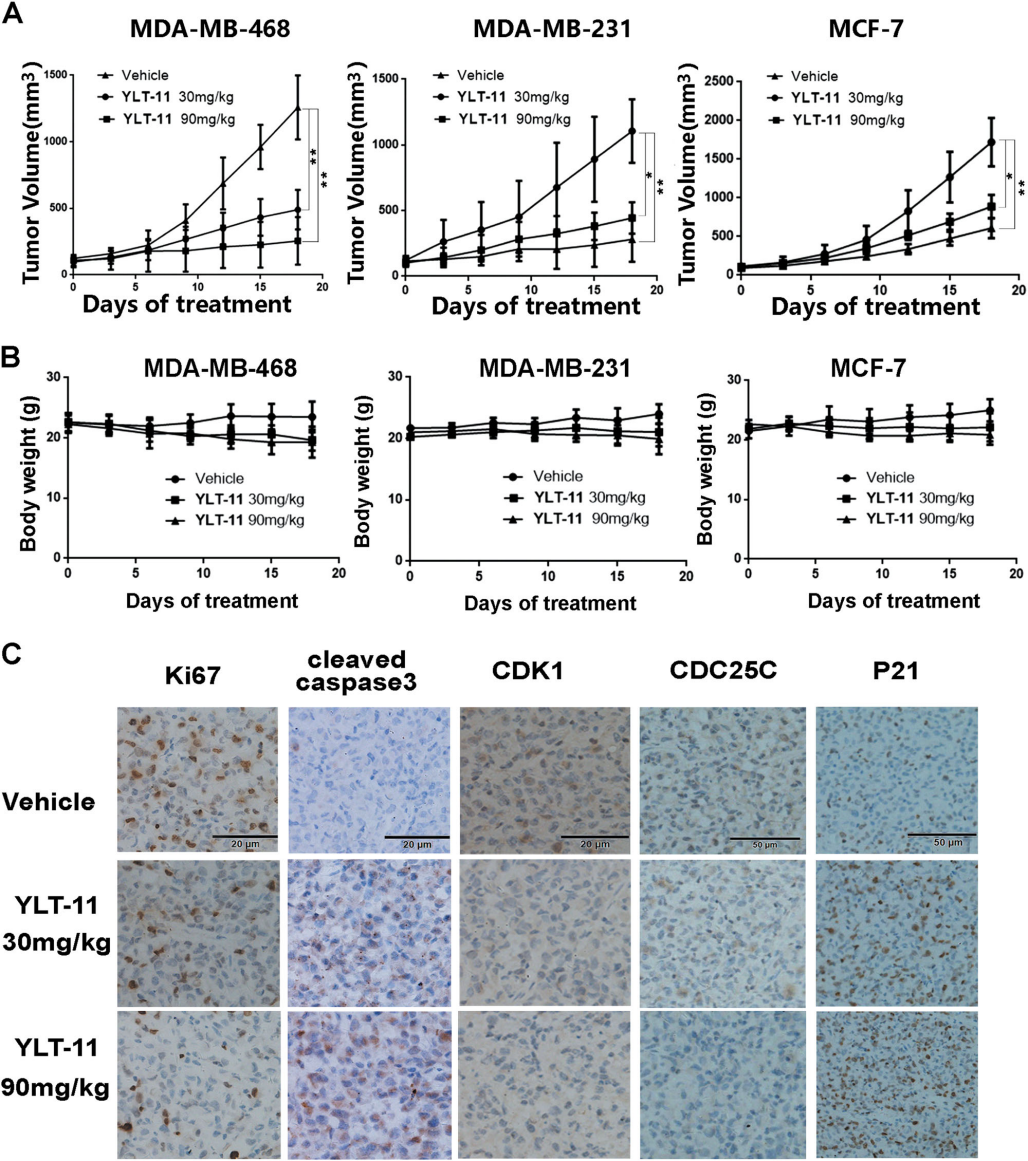
Effects of YLT-11 on the proliferation of xenografts in vivo. **a**, **b** Tumor suppression of MDA-MB-468, MDA-MB-231, and MCF-7 tumor xenografts in mice treated with different concentrations of YLT-11. Tumor size and body weight were measured and calculated every 3 days and presented as mean ± SD (*n* = 5; *p < 0.05; **p < 0.01). **c** Tumor tissues from MDA-MB-231 xenografts treated with vehicle or YLT-11 were immunohistochemically analyzed with anti-Ki67, cleaved caspase-3, and CDC25C, CDK1, and P21

To further evaluate the antitumor activity of YLT-11 in vivo, three breast tumor xenograft models (MCF-7, MDA-MB-468, and MDA-MB-231) were used in this study. Mice bearing subcutaneously implanted tumor were treated with YLT-11 at doses of 30 and 90 mg/kg (p.o.), or blank solvent every day. Notably, YLT-11 remarkably inhibited the growth of tumor xenografts in a dose-dependent manner with tumor inhibition rates of 68, 82.5, and 76.7% at a dosage of 90 mg/kg, respectively, and the final average tumor weight and volume in the treated group were much smaller than those in the vehicle-treated group (Fig. 6a and Supplementary Fig 4a). Most importanty, YLT-11 treatment was well tolerated and caused no significant loss in body weight in experimental animals (Fig. 6b), indicating that YLT-11 was an effective agent for treatment of breast cancer with better tolerability.

To investigate the mechanisms of the antitumor effects of YLT-11 in vivo, immunohistochemical analysis was performed on tumor tissues resected from MDA-MB-231 models. As depicted in Fig. 6c and Supplementary Fig 4b, YLT-11 significantly decreased the number of Ki67-positive cells (from 78 ± 8.1% to 29 ± 6.3%, *p* < 0.01), while the number of cleaved caspase-3-positive cells increased from 12.3 ± 5.1 to 66 ± 7.3% (*p* < 0.01), indicating that YLT-11 could suppress tumor growth in vivo in antiproliferation and pro-apoptosis manner. Recent studies have indicated that PLK4 could activate CDC25C, which controlled the G2–M transition by regulating the CDK1 level^36^. Therefore, we determined the possible effects of YLT-11 on downstream substrates of PLK4 in vivo. YLT-11 effectively suppressed the expression of CDC25C and CDK1 and increased the level of P21. Together, these data revealed that YLT-11 regulated the expression of cell cycle-related proteins in vivo, which in turn inhibited the growth of breast tumor.

**Fig. 7:**
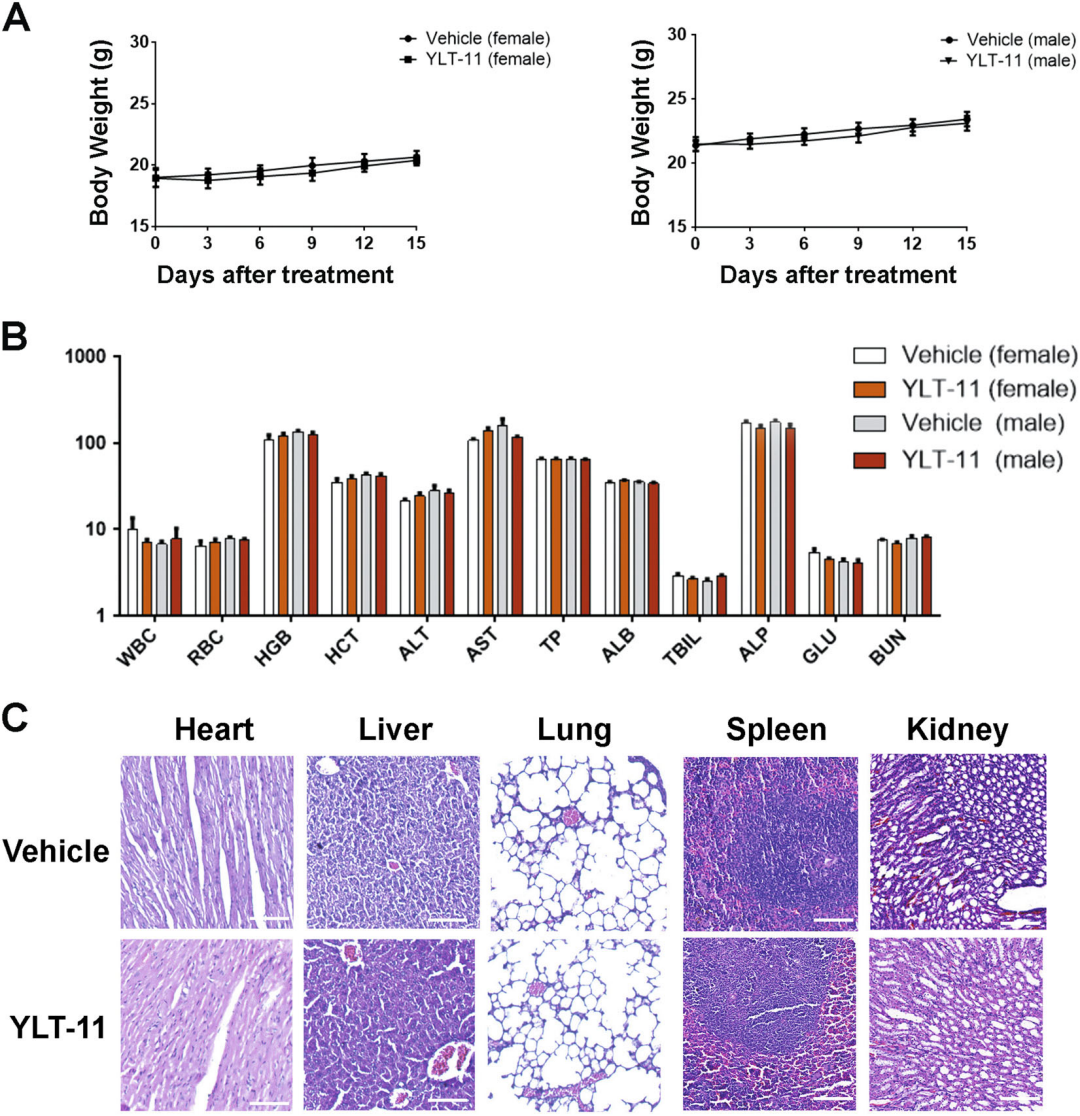
Preliminary safety evaluation of YLT-11 in BALB/c mice. **a** The difference of body weights between two administrated groups (female and male mice) and two vehicle groups (female and male mice) were not significant. Data are expressed as mean ± SD (*n* = 5). **b** Hematological and serum biochemical values of mice at day 14 (*n* = 5) for both vehicle and treated groups. Units of the parameters are as follows: WBC white blood cell (10^9^/L); RBC red blood cell (10^12/^L); HGB hemoglobin (g/L); ALB albumin and TP total protein (g/L); ALT alanine transarninase (U/L); AST aspartate aminotransferase (U/L); TBIL total bilirubin (μmol/L); ALP alkaline phosphatase (U/L); BUN blood urea nitrogen; and GLU glucose (mM). **c** YLT-11 did not cause obvious pathologic abnormalities in normal tissues. Paraformaldehyde-fixed organs (heart, liver, spleen, lungs, and kidneys) were processed for paraffin embedding and then stained by hematoxylin and eosin. Images shown are representatives from each group. Scale bars, ×20 for micrograph

Furthermore, results from sub-acute toxicity study in mice after treatment with YLT-11 indicated that no obvious difference was observed in body weight and the hematoxylin and eosin staining between the YLT-11 treatment and the vehicle treatment groups (Fig. 7a, b). Besides, there was also no significant difference observed in the serological and hematological analyses (Fig. 7c).

## Discussion

PLK4 is an important cell cycle regulator that plays a vital role in centrosome duplication and mitotic progression. Dysregulation (both hyperactivation and deactivation) of PLK4 predisposes cells to the development of cancer^37^. In recent years, numerous studies have reported that PLK4 is hyperactivated in several kinds of human cancers, including breast cancer, colorectal cancer, and pancreatic cancer^16,38^. Suppression of PLK4 activity may offer a novel strategy for human cancer therapy. However, to date, studies about PLK4 inhibitors are limited and only one inhibitor is undergoing phase I clinical trial. Here, we present a novel PLK4 inhibitor, YLT-11, and further investigate the functional characterization and the possible mechanism against human breast cancer.

Kinase activity assays indicated that YLT-11 showed potential inhibition of PLK4 enzymatic activity and exhibited >200-fold selectivity for PLK4 over other PLK family members (PLK1, PLK2, and PLK3) in vitro. Molecular docking suggested that YLT-11 could fit well into the ATP pocket of PLK4, which enables it to be a potent PLK4 inhibitor. Moreover, YLT-11 possessed the ability to inhibit a range of human breast cancer cell lines, especially  for the TNBC cell lines, whereas it exhibited weak inhibitory activity to the normal mammary cell. This is a potentially important finding, in that YLT-11 might provide an alternative method for the treatment of TNBC, of which currently no target drug is approved to use for the treatment in clinic.

Centrosome is an important organelle for mitotic cycle in eukaryotic cells. Abnormal duplication of centrosome is able to result in abnormalities in spindle function and mitotic arrest, subsequently leading to cell death^39–41^, which might be a key mechanism of YLT-11 to exert its anticancer activity. In our study, YLT-11 blocked the activity of PLK4 and induced abnormal centriole duplication. We found that YLT-11 caused an increase in centriole number at low inhibitory concentrations (≤0.25 μM) and a decrease at high concentrations (≥0.5 μM). This phenomenon might be ascribed to the different outcomes between full and partial suppression of PLK4 activity. And increasing the expression of PLK4 both in MDA-MB-468 and MDA-MB-231 at low concentrations of YLT-11 supported this assumption. It was reported that amplification or inhibition of centriole numbers could lead to asymmetric cell division in the next round of mitosis cycle and ultimately trigger mitotic catastrophe^42,43^. We found that YLT-11 initiated a series of characteristics about mitotic catastrophe, including multinucleation, micronuclei, and chromosome mal-disjunction, resulting in the accumulation of aneuploidy/polyploidy and genomic instability. Recent studies showed that aneuploidy makes cancer cells more sensitive to cancer therapy^44^. Those rampant aneuploid cells would cause progressive failure of breast cancer cell division after undergoing several cell cycles, ultimately inducing cell death.

The eukaryotic mitotic cycle consists of a battery of molecular events that are orderly controlled by multiple checkpoint proteins. Cell cycle arrest is induced by abnormalities in spindle function and initiate mitotic slippage/catastrophe by destruction of mitotic checkpoint proteins, resulting in aneuploid and micronucleus cells^45–47^. On the other hand, recent studies have well documented that PLK4 could activate BubR1, which peaked in mitosis and decreased when the cells exited mitosis. We thus speculated that YLT-11-induced dysregulation of cell cycle progression might be due to the effect of PLK4 on downstream mitotic checkpoint kinase. Our results manifested that a prometaphase delay was induced by YLT-11 through prolonging the expression of cyclinB1/CDK1 at a high level in nocodazole-synchronized MDA-MB-468 cells. And then BubR1 destruction was triggered in arrested mitotic cells by YLT-11, which caused mitotic exit without proper segregation of sister chromatids and cytokinesis. Moreover, an increase in cleaved caspase-3 expression and Annexin V/PI-positive staining was observed with treatment of MDA-MB-468 cells, indicating that cells which exited the mitotic phase aberrantly were unable to deal with the demands of continuous proliferation, ultimately leading to mitotic catastrophe and rapid apoptotic death.

These cell data prompt further investigation into the anticancer effects of YLT-11 on breast xenograft models. YLT-11 displayed conspicuous inhibitory effects on tumor growth in breast xenograft models, especially in TNBC models, and the inhibitory rate was more than 60% at the dose of 90 mg/kg (p.o), with no obvious toxicity. Meanwhile, studies of mechanism of action indicated that YLT-11 inhibited tumor cell proliferation and induced apoptosis in vivo, which were mainly through the inhibition of cell cycle-associated proteins.

In summary, we reported here that YLT-11 is a novel and specific small-molecule inhibitor in cancer therapy with a mechanism of action that involves valid suppression of PLK4 activity. YLT-11 has remarkably antiproliferative activity in vitro and antineoplastic activity in human breast cancer xenograft without causing any significant toxicity. Collectively, all the data provided here corroborate that YLT-11 could be a promising lead compound for the treatment of breast cancer and deserves further studies.

## Materials and methods

### Preparation of YLT-11

YLT-11 formulated as (*E*)-*N*-(4-(3-(4-((dimethylamino)methyl)styryl)-1*H*-indazol-6-yl)pyrimidin-2-yl)acetamide was synthesized at the State Key Laboratory of Biotherapy, Sichuan University (Sichuan, China)^32^. YLT-11 was dissolved in dimethyl sulfoxide (DMSO) at a stock concentration of 20 mM and diluted to the appropriate concentration in vitro assay, while in vivo study, YLT-11 was suspended in PEG400:Water (30:70).

### Cell lines and cell culture condition

Human cancer cell lines MDA-MB-468 was purchased from the Type Culture Collection of Chinese Academy of Science (Kunming, China). All the other cell lines were obtained from the American Type Culture Collection (ATCC, Manassas, VA, USA). The cell lines were cultured in RPMI-1640 or Dulbecco's modified Eagle's medium containing 10% fetal bovine serum (v/v), 4 mM l-glutamine, 100 U/mL penicillin, and 0.1 mg/mL streptomycin according to the guidelines of the manufacturer and then incubated in a humidified atmosphere under 5% CO_2_ at 37 °C.

### Molecular docking simulations

The AutoDock Vina program^48^ was applied for molecular docking studies. The compound YLT-11 was prepared as pdbqt file, using AutoDock Tools. The crystal structure of PLK4 complexed with the inhibitor 400631 was downloaded from the PDB database (PDB ID: 4JXF). All the water and solvent molecules were removed. Gasteiger–Marsili charges were added to the protein model, and non-polar hydrogens were then merged onto their respective heavy atoms^49^. The grid center was set as coordinates of *x*, *y*, *z* = 26.375, −20.547, −47.589, and the grid size was 25 Å × 25 Å × 25 Å, which encompasses the ATP-binding pocket. The other parameters for Vina were set as default. The docking results were viewed using PyMol program.

### Western blot

The Western blot assay was performed as described previously^50^. Briefly, cell lysates from breast cancer cell were centrifuged and protein concentrations were measured. Next, proteins from each sample were separated by sodium dodecyl sulfate-polyacrylamide gel electrophoresis (SDS-PAGE) gels and transferred electrophoretically onto polyvinylidene difluoride membrane. Then, the membranes were incubated with appropriate primary antibody and the corresponding secondary antibody. Specific protein bands were detected via chemiluminescence detection and quantified via ImageJ through three independent experiments.

### Cellular thermal shift assay

The ability of YLT-11 to interact with PLK4 was evaluated as described by Molina et al^51^. Cells were harvested and diluted in phosphate-buffered saline (PBS) supplemented with protease inhibitor cocktail. Cells lysates were extracted with freeze-thawing method and separated from the cell debris by centrifugation at 20,000 × *g* for 20 min at 4 °C. Then, the cell lysates were divided into two aliquots, with one aliquot being treated with YLT-11 (10 μM) and the other aliquot with vehicle. After 20–40 min incubation at room temperature, the respective lysates were divided into smaller (50 µL) aliquots and heated individually at different temperatures for 3–5 min, followed by cooling at room temperature for 3 min. The appropriate temperatures were determined in preliminary cellular thermal shift assay experiments. The heated lysates were centrifuged at 20,000 × *g* for 30 min at 4 °C in order to separate the soluble fractions from precipitates. The supernatants were transferred to new microtubes and analyzed by SDS-PAGE, followed by western blot analysis.

### Binding constant assays

Kinase-tagged T7 phage strains were prepared in an *Escherichia coli* host derived from the BL21 strain. *Escherichia coli* were grown to log phase and infected with T7 phage and incubated with shaking at 32 °C until lysis. The lysates were centrifuged and filtered to remove cell debris. Kinases were produced in HEK-293 cells and subsequently tagged with DNA for quantitative polymerase chain reaction (qPCR) detection. Streptavidin-coated magnetic beads were treated with biotinylated YLT-11 for 30 min at room temperature to generate affinity resins for kinase assays. The beads were blocked with excess biotin and washed with blocking buffer (SeaBlock, 1% bovine serum albumin, 0.05% Tween-20, 1 mM dithiothreitol (DTT)). Binding reactions were assembled by combining kinases, ligand affinity beads, and test compounds in 1× binding buffer (20% SeaBlock, 0.17× PBS, 0.05% Tween-20, 6 mM DTT). *K*_d_s were determined using an 11-point threefold YLT-11 dilution series with three DMSO control points. All compounds for *K*_d_ measurements are distributed by acoustic transfer (non-contact dispensing) in 100% DMSO. The assay plates were incubated at room temperature with shaking for 1 h, and the affinity beads were washed with wash buffer (1× PBS, 0.05% Tween-20). The beads were then re-suspended in elution buffer (1× PBS, 0.05% Tween-20, 0.5 µM non-biotinylated affinity ligand) and incubated at room temperature with shaking for 30 min. The kinase concentration in the eluates was measured by qPCR.

### Additional in vitro studies

The additional experiments in vitro including cell proliferation and colony formation assay, EdU incorporation assay, siRNA transfection assay, cell cycle and apoptosis analysis, immunofluorescence, and transmission electron microscope analysis are described in Supplementary methods.

### Subcutaneous xenograft models

All animal experiments were approved by the Institutional Animal Care and Treatment Committee of Sichuan University, China (Permit Number: 20160188), and were carried out in accordance with the approved guidelines. The detailed experimental operations are described in Supplementary methods.

### Statistical analysis

Statistical analyses were carried out in Microsoft Excel and GraphPad Prism 6.0. The statistical significance of results in all the experiments was examined by Student’s *t* test and analysis of variance. *P* value which is <0.05 was defined as statistically significant.

## Electronic supplementary material

supplementary method and figure

supplementary table 1

supplementary table 2
