# Supplementary material for: YLT-11, a novel PLK4 inhibitor, inhibits human breast cancer growth via inducing maladjusted centriole duplication and mitotic defect
Source: Cell Death Dis. 2018 Oct 18;9(11):1066. doi: 10.1038/s41419-018-1071-2 (PMC6194023; doi:10.1038/s41419-018-1071-2)
Supplement: Supplementary file 1 — supplementary method and figure [file 41419_2018_1071_MOESM1_ESM.docx]

**Supplementary Methods**

**Cell proliferation and** **colony formation assay**

The human breast cancer cell lines seeded in 96-well plates were treated with an escalating doses of YLT-11 for indicated times, then the ability of cell proliferation was determined via using an MTS assay that the optical density was measured using a Spectra max M5 microplate spectrophotometer (Molecular Devices) at 490 nm. The IC_50_ values were calculated using the GraphPad Prism software, each assay was replicated 3 times.

The colony formation assay was performed as described previously. Briefly, cells were seeded in 6-well plates and treated with indicated concentrations, stained by 0.5% crystal violet solution after washed with PBS and fixed with methanol. The colonies were counted to assess the inhibition of cell colony formation potential.

**The EdU incorporation assay**

EdU can incorporate into replicating DNA when the cells are dividing, and it isused to label proliferating cells. MDA-MB-468 and MDA-MB-231 cell lines (5000/well) were seeded in 96-well plates and treated with indicated concentration of YLT-11 for 24 h, and then the Cell-Light^TM^ EdU DNA Cell Proliferation Kit was used as the description of the manufacture’s instruction to assess the level of incorporation. Each assay was replicated 3 times.

**siRNA transfection**

The human mammary carcinoma cell lines were seeded in the 96-well or 6-well plate for 24 h, Transient transfection was performed using Lipofectamine2000 or LipofectamineRNAiMAX (Invitrogen) according to manufacturer’s instructions. Cells were transfected with validated siRNA for PLK4 at a concentration of 50~100 nM. The siRNA kits were purchased from RiboBio (Guangzhou, China).

**qRT-PCR**

RNAs were reverse-transcribed into cDNA with the Reverse Transcription System, according to the manufacturer's protocol (Mei 5 Biotechnology, Co., Ltd). PLK4 gene expression was quantified by Real-time RT-PCR, the  experiments were performed using SYBR Green Supermix (BIO-RAD) and the following primers: hPLK4  forward: 5’ – CTCAGTGGGGAAATCAAGAA – 3’, hPLK4 reverse: 5’ – CTCAGTGGGGAAATCAAGAA – 3’; hβ-actin forward: 5’- CATGTACGTTGCTATCCAGGC – 3’, hβ-actin reverse: 5’ - CTCCTTAATGTCACGCACGAT– 3’.

**Immunofluorescence**

Tumor cells were seeded in Millicell EZ SLIDE 8 well glass slide (Millipore, Ireland) at the density of 2 × 10^4^ per well. The next day, different concentration of YLT-11 was added to treat the cell lines for 24 h. cells were washed twice in PBS, fixed in 4% paraformaldehyde for 15 min and washed three times at 3 min intervals. Then cells were incubated with primary antibody overnight at 4 ℃ after blocked with 1% BSA. Goat anti-rabbit and goat anti-mouse secondary antibodies conjugated to FITC and Alexa 598 were used to detect primary antibodies. Cell nucleuses were stained with DAPI. Images were taken using a Leica cofocusing microscope.

**Transmission electron microscope**

MDA-MB-468 and MDA-MB-231 cell lines (10^6^ /well) were seeded in 6-well plates and treated with indicated concentrations of YLT-11 for 24 h, then cells were harvested and fixed with 2.5% pre-cooling glutaraldehyde overnight. After washed with PBS for three times, cells were ﬁxed with 1% osmium tetroxide and dehydrated in different concentration of acetone, followed by embedding in Eponate-12 resin. 50–70 nm sections were cut parallel to monolayer surface, counter stained with 4% aqueous uranyl acetate. Then the results were observed on the transmission electron microscope.

**Flow** **cytometry assay**

To determine the aneuploid cells, breast cancer cells were treated with YLT-11 for indicated time and harvested into polypropylene tubes. The cells were washed with PBS and fixed in 75% ethanol at -20 ℃ overnight followed by incubation with propidium iodide (PI) for 15 min at room temperature. Then DNA content per cell was determined by flow cytometry (FCM). Data were analyzed using the NovoExpress and Flow Jo software.

For the apoptosis analysis, the Annexin Ⅴ-FITC apoptosis detection kit was used as the manufacturer’s protocol. Briefly, harvested cells were stained with Annexin Ⅴ-FITC and PI and incabated for 15 min in dark. Then the stained cells were detected by FCM and analyzed with the NovoExpress and Flow Jo software.

**Hoechst 33342 staining for morphological analysis of nuclei**

Cells in the six-well plates were treated with YLT-11 at different concentration for 30 h. Then treated tumor cells were washed with PBS and stain with Hoechst 33342 in the dark for 15 min. Image were taken by fluorescence microscopy for morphological changes after YLT-11 treatment.

**Immunohistochemistry**

Tumor tissues obtained from the animal models were immersed in 4% paraformaldehyde. After washing with running tap water overnight, the tissues dehydrated trough a serial alcohol gradient and embedded in paraffin, Paraffin-embedded tumor sections were stained with primary antibodies (Ki67, cleaved caspase-3, p21, CDC25C, CyclinB1) using the DAB Detection Kit. The images were captured with a Carl Zeiss digital camera attached to a light microscope.

**Subcutaneous xenograft models**

For the MCF-7 tumor xenograft, six-week-old female BALB/c nude mice were given 100 μL cell suspension (about 4×10^6^ tumor cells) subcutaneous injection on the right flank and the mice was randomly assigned into three group (n=5) after the average volume of the tumor reached to 100~150 mm^3^, then indicated doses of YLT-11 and vehicle were administered once daily by oral gavage.

For MDA-MB-231 and MDA-MB-468, firstly, we obtain the donor tumor by subcutaneously injecting 100 μL cell suspension containing 1~2×10^7^ cells into the female BALB/c nude mice on the right flank. Then the tumor was take out from the mice and dissected into fragment of about 15~20 mm^3^ in the culture medium without serum and antibiotics. Subsequently, the tiny tumor tissues were subcutaneously implanted into the BALB/c nude mice, when the tumor reached to about 100 mm^3^, YLT-11 or blank solvent were administered orally. For all subcutaneous xenograft models, size of tumor and weight of mice were monitored every 3 days, the tumor volume were calculated as the following the formula: tumor volume (mm^3^) =0.5×L×W^2^(L represents length and W represents width).

**Sub-acute toxicity test**

A sub-toxicity test was performed in healthy BALB/c mice which were orally administrated with a single dose of 1 g/kg of YLT-11.The clinical symptoms of the mice, including mortality and body weights, were observed for 15 days. Then blood of the mice was obtained for serum biochemistry and hematological analysis using a Hitachi 7200 Blood Chemistry Analyzer and a Nihon Kohdern MEK-5216K Automatic Hematology Analyzer. The heart, liver, spleen, lung and kidney were fixed and routinely processed and embedded in paraﬃn for hematoxylin and eosin (H&E) staining analysis.

**Supplementary Figures**


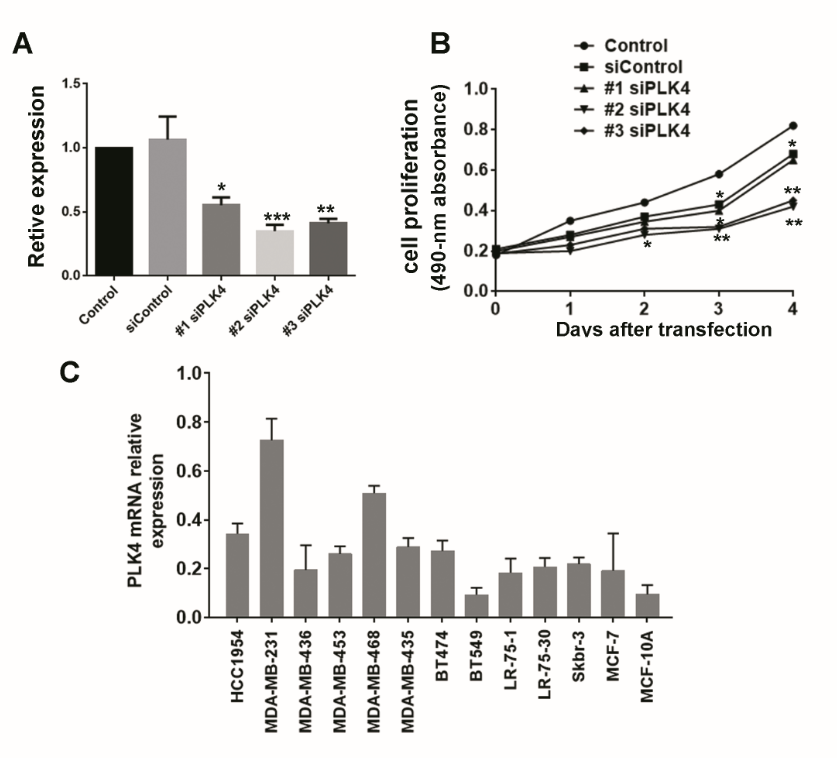


**Supplementary Figure 1. The effects of PLK4 expression on breast cancer cell growth. (A)** Expression of PLK4 in MDA-MB-231 cell transfected with PLK4 siRNA was quantified by the densitometry analysis using Image J. Data are expressed as mean ± SD for 3 independent experiments. (B) Breast cancer cell deﬁcient in PLK4 protein expression exhibited decreased proliferation. Anchorage-dependent cell growth was determined at 1, 2, 3 and 4 days using the MTS assay. (C) Expression of PLK4 in different types of breast cancer cells and normal breast cell. β-actin mRNA level was used for data normalization. Data are expressed as mean ± SD for 3 independent experiments. Columns, mean; bars, SD, (*p < 0.05, **p < 0.01, ***p < 0.001 vehicle control).


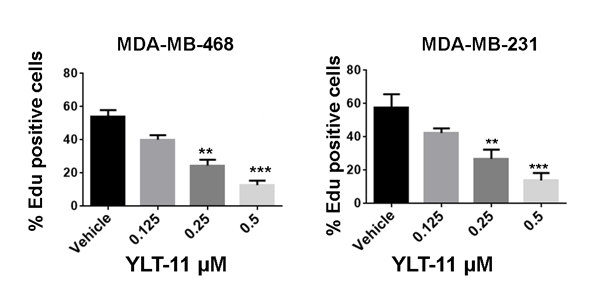


**Supplementary Figure 2. YLT-11 inhibited breast cancer cell proliferation.** Edu positive staining of MDA-MB-468 and MDA-MB-231 were quantification.


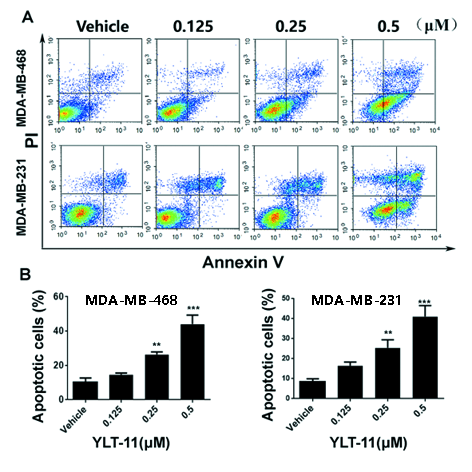


**Supplementary Figure 3. YLT-11 induced apoptosis of cancer cells. (A and B)** The effects of YLT-11 on inducing MDA-MB-468 and MDA-MB-231 cells apoptosis were analyzed after treated with the indicated concentrations of YLT-11 and DMSO for 24 hours. Data are expressed as mean ± SD for 3 independent experiments. Columns, mean; bars, SD, (*p < 0.05, **p < 0.01, ***p < 0.001 vehicle control).


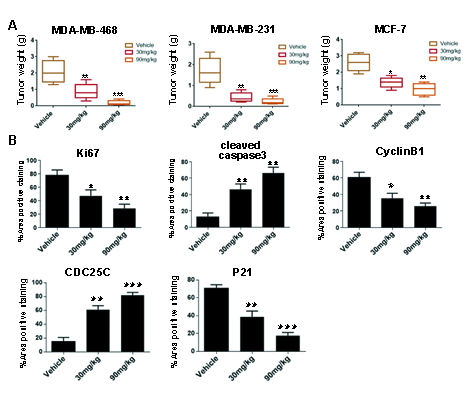


**Supplementary Figure 4. Antitumor efficacy of YLT-11 in vivo.** (A) Represented weight of tumors from mice in different groups. Data are presented as mean ± SD (n = 5; *P<0.05; **P<0.01; ***p < 0.001 vehicle control). (B) quantification of immunohistochemical analysis. P values for comparison of two groups were determined by 2-tailed Student’s t test (*P < 0.05; **P < 0.01; ***P < 0.001 vs vehicle control).
